# *meso*-4,4′-Difluoro-2,2′-{[(3a*R*,7a*S*)-2,3,3a,4,5,6,7,7a-octa­hydro-1*H*-1,3-benzimidazole-1,3-di­yl]bis­(methyl­ene)}diphenol

**DOI:** 10.1107/S1600536813000305

**Published:** 2013-01-12

**Authors:** Augusto Rivera, Diego Quiroga, Jaime Ríos-Motta, Monika Kučeraková, Michal Dušek

**Affiliations:** a Facultad de Ciencias, Departamento de Química, Universidad Nacional de Colombia, Sede Bogotá, Cra 30 No. 45-03, Bogotá, Código Postal 111321, Colombia; b Institute of Physics, Academy of Sciences of the Czech Republic, v.v.i., Na Slovance 2, 182 21 Praha 8, Czech Republic

## Abstract

In the crystal structure of the title compound, C_21_H_24_F_2_N_2_O_2_, there are two intra­molecular O—H⋯N hydrogen bonds involving the N atoms of the imidazolidine ring and the hy­droxy groups. The crystal studied was a *meso* compound obtained by the reaction of the aminal (2*S*,7*R*,11*S*,16*R*)-1,8,10,17-tetra­aza­penta­cyclo­[8.8.1.1^8,17^.0^2,7^.0^11,16^]cosane with 4-fluoro­phenol. The imidazolidine ring has a twisted conformation with a CH—CH—N—CH_2_ torsion angle of 44.99 (14)° and, surprisingly, the lone pairs of the N atoms are disposed in a *syn* isomerism, making the title compound an exception to the typical ‘rabbit-ear effect’ in 1,2-diamines. In the crystal, molecules are linked *via* C—H⋯F hydrogen bonds, forming chains along the *c*-axis direction. These chains are linked *via* another C—H⋯F hydrogen bond, forming a three-dimensional network.

## Related literature

For a related structure, see: Rivera *et al.* (2011 ▶). For a discussion of the ‘rabbit-ear effect’ in 1,2-diamines, see: Hutchins *et al.*(1968 ▶).
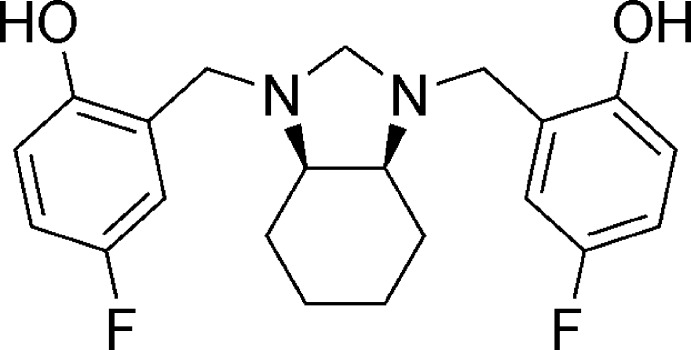


## Experimental

### 

#### Crystal data

C_21_H_24_F_2_N_2_O_2_
*M* *_r_* = 374.4
Orthorhombic, 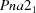

*a* = 15.4029 (4) Å
*b* = 18.7822 (4) Å
*c* = 6.1639 (2) Å
*V* = 1783.22 (8) Å^3^
*Z* = 4
Cu *K*α radiation
μ = 0.86 mm^−1^
*T* = 120 K
0.31 × 0.15 × 0.11 mm

#### Data collection

Agilent Xcalibur (Atlas, Gemini ultra) diffractometer
Absorption correction: multi-scan (*CrysAlis PRO*; Agilent, 2010 ▶) *T*_min_ = 0.222, *T*_max_ = 1
40310 measured reflections
3177 independent reflections
2984 reflections with *I* > 3σ(*I*)
*R*_int_ = 0.049

#### Refinement

*R*[*F* ^2^ > 2σ(*F* ^2^)] = 0.030
*wR*(*F* ^2^) = 0.077
*S* = 1.42
3177 reflections
250 parameters
H atoms treated by a mixture of independent and constrained refinement
Δρ_max_ = 0.15 e Å^−3^
Δρ_min_ = −0.11 e Å^−3^

### 

Data collection: *CrysAlis PRO* (Agilent, 2010 ▶); cell refinement: *CrysAlis PRO*; data reduction: *CrysAlis PRO*; program(s) used to solve structure: *SUPERFLIP* (Palatinus & Chapuis, 2007 ▶); program(s) used to refine structure: *JANA2006* (Petříček *et al.*, 2006 ▶); molecular graphics: *DIAMOND* (Brandenburg & Putz, 2005 ▶); software used to prepare material for publication: *JANA2006*.

## Supplementary Material

Crystal structure: contains datablock(s) global, I. DOI: 10.1107/S1600536813000305/bx2435sup1.cif

Structure factors: contains datablock(s) I. DOI: 10.1107/S1600536813000305/bx2435Isup2.hkl

Supplementary material file. DOI: 10.1107/S1600536813000305/bx2435Isup3.cml

Additional supplementary materials:  crystallographic information; 3D view; checkCIF report

## Figures and Tables

**Table 1:** Hydrogen-bond geometry (Å, °)

*D*—H⋯*A*	*D*—H	H⋯*A*	*D*⋯*A*	*D*—H⋯*A*
O1—H1⋯N1	0.85 (2)	1.89 (2)	2.6540 (17)	148 (2)
O2—H2⋯N2	0.85 (2)	1.89 (2)	2.6741 (18)	152 (2)
C13—H1C13⋯F2^i^	0.96	2.43	3.2645 (19)	145
C17—H2C17⋯F2^ii^	0.96	2.54	3.356 (2)	142

Symmetry codes: (i) 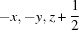; (ii) 
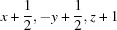
.

**Fig. 1.**
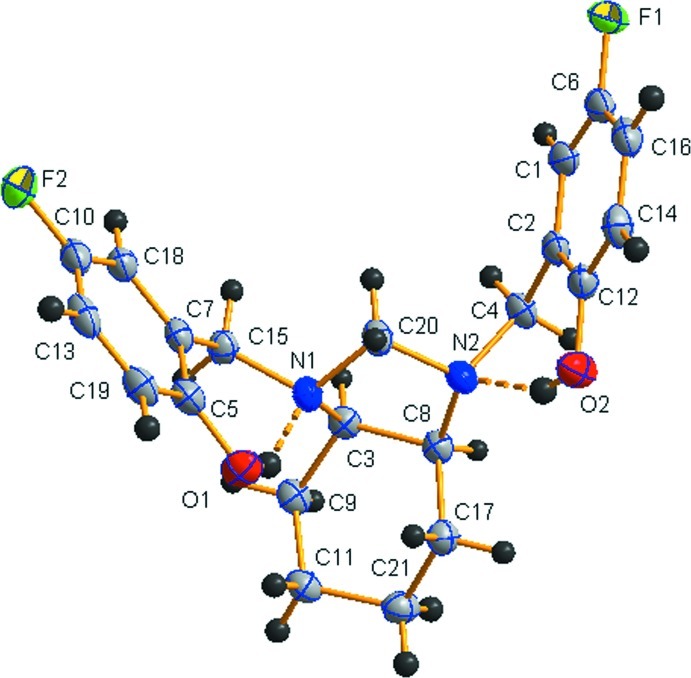
A perspective view of the title compound, showing the atom-numbering scheme. Displacement ellipsoids are drawn at the 50% probability level and H atoms are shown as small spheres of arbitrary radii. Hydrogen bonds are drawn as dashed lines.

## supplementary crystallographic information

### Comment

Typically the 1,1-diamines tend to adopt a conformation in which the arrangement of electron pairs is *anti* periplanar. This behavior is known as `rabbit-ears' effect (Hutchins *et al.*, 1968), however, this effect can be avoided by restriction of the 1,2-diamine in cyclic molecules. Recently, we reported the synthesis and the crystal structure of *rac*-4,4'-difluoro-2,2'-{[(3a*RS*,7a*RS*)-2,3,3a,4,5,6,7,7a- octahydro- 1*H*-1,3-benzimidazole-1,3-diyl] *bis*(methylene)]}diphenol (Rivera *et al.*, 2011), which has *trans* stereochemistry in the 1,2-diamine moiety with the lone pairs located in *anti* disposition avoiding the repulsive interactions. Now we reported the synthesis and crystal structure of the *meso* diastereoisomer with absolute configuration (*R*,*S*) where surprisingly the lone pairs of the N atoms are located in *syn* disposition.

The molecular structure and atom-numbering scheme for (I) are shown in Fig. 1. In the molecular structure of the title compound, the two N atoms of the heterocyclic ring interact with the H atoms of the hydroxy groups by intramolecular hydrogen bonds O—H···N, with N···O interatomic distance values around 2.66 Å, as well as the values of C—O and O—H bond lengths are 1.363 (3) Å and 0.83 (3) Å, respectively. The cyclohexane ring adopts a chair conformation while the heterocyclic ring arranged diagonally respect to the cyclohexane ring with dihedral angle between planes of 25.46 (96)°. The heterocyclic ring adopts an envelope conformation according to the value of the N2—C5—N1—C16 torsion angle of -7.91°. Bond angles around the N atoms N1 and N2 show a higher *sp*^3^ character to the N1 and N2 N atoms with pyramidalization involved in the hydrogen bond type interactions [Σ(CNC) N1 = 331.3°, Σ(CNC) N2 = 330.4°]. Moreover, the benzyl groups are located in an unexpected 1,3-diequatorial *syn* arrangement in the heterocyclic ring with dihedral angle between the planes containing the aromatic rings of 53.80 (30)°. The nonbonding pairs of amino groups involved in the intermolecular hydrogen bonding interactions do not suffer the `rabbit-ear effect' having a *syn* arrangement demonstrating that the title compound is an exception of this effect.

The stability of the crystal lattice of the title compound is related with non classical intermolecular interactions C—H···F that hold molecules linked in extended chains along the *c* axis. There are O—H···C and C—H···F weak interactions (table 1), the latter involve halogen group in molecular contact with an electron-deficient C—H bond of the aromatic ring of a second molecule.

### Experimental

To a stirred solution of (2*S*,7*R*,11*S*,16*R*)-1,8,10,17-tetraazapentacyclo[8.8.1.1.^8,17^0.^2,7^0^11,16^] icosane (3) (276 mg, 1.00 mmol) in dioxane (3 ml) was added slowly dropwise *p*-fluorophenol (2.00 mmol) in dioxane (3 ml). After stirring for 15 min at room temperature, water (4 ml) was added and the mixture was heated at 40°C during 30 h. After cooling to room temperature, the solvent was removed *in vacuo* and the crude product was purified by chromatography on a silica column and subjected to gradient elution with light petroleum ether:ethyl acetate (yield 20%, m.p. = 441–443 K). Single crystals of (I) were grown from a CHCl_3_ solution by slow evaporation of the solvent at room temperature over a period of about 2 weeks. FT–IR (KBr) ν_max_: 3061, 2848, 1495, 1448, 1387, 1289, 1245, 1194, 1124, 1063, 979, 925, 814, 772, 737, 714, 692, 668 cm^-1. 1^H NMR (400 MHz, CDCl_3_) δ (p.p.m.): 1.36 (*m*, 2H), 1.55–1.79 (*m*, 6H), 3.11 (*t*, 2H, *J*_H,H_ = 4.0 Hz), 3.39 (*d*, 1H,^2^*J*_H,H_ = 6.4 Hz, NCH_2_N), 3.63 (*d*, 2H, ^2^*J*_H,H_ = 14.0 Hz, ArCH_2_N), 3.84 (*d*, 1H, ^2^*J*_H,H_ = 6.4 Hz, NCH_2_N), 4.03 (*d*, 2H, ^2^*J*_H,H_ = 14.0 Hz, ArCH_2_N), 6.70 (*dd*, 2H, ^3^*J*_H,F_ = 8.0 Hz, ^4^*J*_H,H_ = 2.8 Hz, Ar—H), 6.76 (*dd*, 2H, ^3^*J*_H,H_ = 8.0 Hz, ^4^*J*_H,F_ = 4.8 Hz, Ar—H), 6.87 (*td*, 2H, ^3^*J*_H,H_ = 8.0 Hz, ^3^*J*_H,F_ = 8.2 Hz, ^4^*J*_H,H_ = 3.1 Hz, Ar—H), 10.34 (*s*, 2H). ^13^C NMR (100 MHz, CDCl_3_) δ (p.p.m.): 21.5, 24.7, 55.0, 61.1, 73.4, 114.7 (d, ^2^*J*_H,F_ = 23.5 Hz), 115.4 (d, ^2^*J*_H,F_ = 22.5 Hz), 117.0 (d, ^3^*J*_H,F_ = 6.3 Hz), 122.0 (d, ^3^*J*_H,F_ = 6.9 Hz), 153.4 (d, ^4^*J*_H,F_ = 2.0 Hz), 156.0 (d, ^1^*J*_H,F_ = 236 Hz).

### Refinement

All H atoms were discernible in difference Fourier maps and could be refined to reasonable geometry. According to common practice H atoms bonded C atoms were kept in ideal positions with C—H distance 0.96 Å during the refinement. The hydroxy H atoms were found in difference Fourier maps and their coordinates were refined freely. All H atoms were refined with thermal displacement coefficients *U*_iso_(H) set to 1.5*U*_eq_(C, O) for hydroxy groups and to 1.2*U*_eq_(C) for the CH– and CH_2_– groups.

### Crystal data

**Table:**

C_21_H_24_F_2_N_2_O_2_	*F*(000) = 792
*M**_r_* = 374.4	*D*_x_ = 1.394 Mg m^−^^3^
Orthorhombic, *P**n**a*2_1_	Cu *K*α radiation, λ = 1.5418 Å
Hall symbol: P -2ac -2n	Cell parameters from 19583 reflections
*a* = 15.4029 (4) Å	θ = 3.7–67.0°
*b* = 18.7822 (4) Å	µ = 0.86 mm^−^^1^
*c* = 6.1639 (2) Å	*T* = 120 K
*V* = 1783.22 (8) Å^3^	Polygon shape, white
*Z* = 4	0.31 × 0.15 × 0.11 mm

### Data collection

**Table:**

Agilent Xcalibur (Atlas, Gemini ultra) diffractometer	3177 independent reflections
Radiation source: Enhance Ultra (Cu) X-ray Source	2984 reflections with *I* > 3σ(*I*)
Mirror monochromator	*R*_int_ = 0.049
Detector resolution: 10.3784 pixels mm^-1^	θ_max_ = 67.1°, θ_min_ = 3.7°
ω scans	*h* = −18→18
Absorption correction: multi-scan (*CrysAlis PRO*; Agilent, 2010)	*k* = −22→22
*T*_min_ = 0.222, *T*_max_ = 1	*l* = −7→7
40310 measured reflections	

### Refinement

**Table:**

Refinement on *F*^2^	91 constraints
*R*[*F*^2^ > 2σ(*F*^2^)] = 0.030	H atoms treated by a mixture of independent and constrained refinement
*wR*(*F*^2^) = 0.077	Weighting scheme based on measured s.u.'s *w* = 1/(σ^2^(*I*) + 0.0016*I*^2^)
*S* = 1.42	(Δ/σ)_max_ = 0.010
3177 reflections	Δρ_max_ = 0.15 e Å^−^^3^
250 parameters	Δρ_min_ = −0.11 e Å^−^^3^
0 restraints	

### Special details

**Table:**

Refinement. The refinement was carried out against all reflections. The conventional *R*-factor is always based on *F*. The goodness of fit as well as the weighted *R*-factor are based on *F* and *F*^2^ for refinement carried out on *F* and *F*^2^, respectively. The threshold expression is used only for calculating *R*-factors *etc*. and it is not relevant to the choice of reflections for refinement.The program used for refinement, Jana2006, uses the weighting scheme based on the experimental expectations, see _refine_ls_weighting_details, that does not force *S* to be one. Therefore the values of *S* are usually larger than the ones from the *SHELX* program.

### Fractional atomic coordinates and isotropic or equivalent isotropic displacement parameters (Å^2^)

**Table:**

	*x*	*y*	*z*	*U*_iso_*/*U*_eq_	
F1	0.08421 (6)	0.56542 (5)	0.39265 (19)	0.0421 (3)	
F2	0.01983 (7)	0.08785 (5)	−0.0670 (2)	0.0464 (3)	
O1	0.26711 (8)	0.11965 (6)	0.5638 (2)	0.0355 (4)	
O2	0.25923 (8)	0.37737 (7)	0.9132 (2)	0.0369 (4)	
N1	0.33271 (8)	0.22874 (7)	0.3446 (2)	0.0231 (3)	
N2	0.34150 (7)	0.33314 (6)	0.5557 (2)	0.0238 (4)	
C1	0.20351 (10)	0.48809 (8)	0.4427 (3)	0.0283 (5)	
C2	0.24882 (10)	0.44012 (8)	0.5731 (3)	0.0257 (4)	
C3	0.41911 (9)	0.26109 (8)	0.3064 (2)	0.0230 (4)	
C4	0.33424 (10)	0.40923 (8)	0.4988 (3)	0.0270 (4)	
C5	0.20680 (10)	0.11405 (8)	0.4035 (3)	0.0274 (4)	
C6	0.12823 (10)	0.51814 (8)	0.5218 (3)	0.0314 (5)	
C7	0.21420 (10)	0.15102 (8)	0.2071 (3)	0.0254 (4)	
C8	0.42966 (9)	0.30360 (8)	0.5158 (3)	0.0237 (4)	
C9	0.49248 (9)	0.20825 (8)	0.2612 (3)	0.0267 (4)	
C10	0.08186 (11)	0.09680 (8)	0.0901 (3)	0.0324 (5)	
C11	0.52047 (10)	0.16567 (8)	0.4592 (3)	0.0299 (5)	
C12	0.21650 (10)	0.42368 (8)	0.7793 (3)	0.0289 (5)	
C13	0.07318 (10)	0.06018 (9)	0.2813 (3)	0.0343 (5)	
C14	0.13982 (11)	0.45439 (9)	0.8526 (3)	0.0333 (5)	
C15	0.29291 (10)	0.19587 (8)	0.1527 (3)	0.0259 (4)	
C16	0.09529 (11)	0.50248 (9)	0.7232 (3)	0.0340 (5)	
C17	0.45886 (10)	0.25765 (9)	0.7059 (3)	0.0275 (5)	
C18	0.15019 (10)	0.14217 (8)	0.0499 (3)	0.0284 (4)	
C19	0.13591 (10)	0.06908 (8)	0.4391 (3)	0.0331 (5)	
C20	0.28071 (9)	0.28869 (8)	0.4282 (3)	0.0270 (4)	
C21	0.54005 (11)	0.21507 (9)	0.6489 (3)	0.0321 (5)	
H1c1	0.22435	0.499969	0.300447	0.034*	
H1c3	0.422455	0.288956	0.176142	0.0276*	
H1c4	0.339534	0.414724	0.344506	0.0325*	
H2c4	0.381123	0.435124	0.564372	0.0325*	
H1c8	0.473781	0.33942	0.502131	0.0284*	
H1c9	0.541614	0.233235	0.203	0.032*	
H2c9	0.475105	0.176214	0.147852	0.032*	
H1c11	0.475029	0.133249	0.499311	0.0359*	
H2c11	0.571355	0.138428	0.424659	0.0359*	
H1c13	0.024629	0.029077	0.30491	0.0412*	
H1c14	0.117621	0.442274	0.993384	0.0399*	
H1c15	0.335179	0.167023	0.079409	0.0311*	
H2c15	0.276748	0.23231	0.051253	0.0311*	
H1c16	0.042627	0.524324	0.773234	0.0408*	
H1c17	0.412966	0.225597	0.745425	0.033*	
H2c17	0.470585	0.287446	0.828962	0.033*	
H1c18	0.153672	0.167458	−0.08517	0.0341*	
H1c19	0.13078	0.044152	0.574433	0.0397*	
H1c20	0.235882	0.270824	0.521692	0.0324*	
H2c20	0.258396	0.315914	0.308761	0.0324*	
H1c21	0.585954	0.24712	0.60938	0.0386*	
H2c21	0.557641	0.187359	0.772163	0.0386*	
H1	0.3046 (14)	0.1501 (12)	0.523 (4)	0.0426*	
H2	0.2959 (14)	0.3567 (12)	0.832 (4)	0.0443*	

### Atomic displacement parameters (Å^2^)

**Table:**

	*U*^11^	*U*^22^	*U*^33^	*U*^12^	*U*^13^	*U*^23^
F1	0.0366 (5)	0.0315 (5)	0.0583 (7)	0.0066 (4)	−0.0117 (5)	0.0030 (5)
F2	0.0397 (5)	0.0393 (5)	0.0602 (7)	−0.0079 (4)	−0.0155 (5)	−0.0076 (5)
O1	0.0400 (6)	0.0352 (6)	0.0312 (6)	−0.0068 (5)	−0.0009 (5)	0.0091 (5)
O2	0.0452 (7)	0.0398 (7)	0.0259 (6)	0.0104 (5)	0.0049 (6)	0.0029 (5)
N1	0.0233 (6)	0.0226 (6)	0.0234 (6)	−0.0014 (5)	0.0034 (5)	−0.0016 (5)
N2	0.0231 (6)	0.0199 (6)	0.0285 (7)	0.0004 (5)	0.0028 (5)	−0.0003 (5)
C1	0.0308 (8)	0.0214 (7)	0.0328 (9)	−0.0035 (6)	−0.0036 (7)	0.0010 (6)
C2	0.0293 (7)	0.0185 (7)	0.0292 (8)	−0.0026 (6)	−0.0007 (7)	−0.0035 (6)
C3	0.0240 (7)	0.0227 (7)	0.0224 (8)	−0.0007 (6)	0.0037 (6)	0.0024 (6)
C4	0.0304 (7)	0.0203 (7)	0.0304 (9)	−0.0002 (6)	0.0035 (6)	0.0009 (6)
C5	0.0292 (7)	0.0220 (7)	0.0311 (8)	0.0018 (6)	0.0045 (7)	0.0006 (6)
C6	0.0294 (8)	0.0208 (7)	0.0441 (10)	−0.0006 (6)	−0.0077 (7)	−0.0044 (7)
C7	0.0266 (7)	0.0200 (7)	0.0295 (8)	0.0028 (6)	0.0035 (6)	−0.0041 (6)
C8	0.0223 (6)	0.0221 (7)	0.0266 (8)	−0.0011 (6)	0.0025 (6)	−0.0003 (6)
C9	0.0246 (7)	0.0288 (8)	0.0267 (8)	0.0015 (6)	0.0063 (6)	−0.0011 (6)
C10	0.0284 (8)	0.0241 (7)	0.0449 (11)	0.0006 (6)	−0.0021 (7)	−0.0076 (7)
C11	0.0287 (7)	0.0280 (7)	0.0331 (9)	0.0055 (6)	0.0031 (6)	−0.0009 (7)
C12	0.0339 (8)	0.0249 (8)	0.0278 (9)	0.0003 (6)	0.0001 (7)	−0.0046 (6)
C13	0.0287 (8)	0.0221 (8)	0.0522 (11)	−0.0038 (6)	0.0107 (8)	−0.0073 (7)
C14	0.0363 (8)	0.0314 (8)	0.0321 (9)	−0.0007 (6)	0.0069 (7)	−0.0081 (7)
C15	0.0290 (7)	0.0248 (7)	0.0239 (8)	−0.0014 (6)	0.0009 (6)	−0.0004 (6)
C16	0.0289 (7)	0.0273 (8)	0.0459 (11)	−0.0001 (7)	0.0012 (7)	−0.0109 (7)
C17	0.0279 (7)	0.0304 (8)	0.0241 (8)	0.0021 (6)	0.0024 (6)	−0.0012 (6)
C18	0.0324 (8)	0.0224 (7)	0.0306 (9)	0.0005 (6)	0.0000 (7)	−0.0033 (7)
C19	0.0354 (8)	0.0234 (7)	0.0405 (10)	−0.0006 (6)	0.0102 (8)	0.0017 (7)
C20	0.0247 (7)	0.0241 (7)	0.0323 (9)	−0.0002 (6)	0.0033 (7)	−0.0036 (7)
C21	0.0299 (7)	0.0364 (9)	0.0302 (9)	0.0076 (7)	0.0000 (7)	0.0005 (7)

### Geometric parameters (Å, º)

**Table:**

F1—C6	1.372 (2)	C8—C17	1.523 (2)
F2—C10	1.370 (2)	C8—H1c8	0.96
O1—C5	1.360 (2)	C9—C11	1.522 (2)
O1—H1	0.85 (2)	C9—H1c9	0.96
O2—C12	1.368 (2)	C9—H2c9	0.96
O2—H2	0.85 (2)	C10—C13	1.371 (3)
N1—C3	1.4818 (18)	C10—C18	1.377 (2)
N1—C15	1.468 (2)	C11—C21	1.523 (2)
N1—C20	1.4747 (19)	C11—H1c11	0.96
N2—C4	1.4758 (19)	C11—H2c11	0.96
N2—C8	1.4874 (18)	C12—C14	1.390 (2)
N2—C20	1.4805 (19)	C13—C19	1.381 (2)
C1—C2	1.395 (2)	C13—H1c13	0.96
C1—C6	1.379 (2)	C14—C16	1.387 (2)
C1—H1c1	0.96	C14—H1c14	0.96
C2—C4	1.509 (2)	C15—H1c15	0.96
C2—C12	1.400 (2)	C15—H2c15	0.96
C3—C8	1.526 (2)	C16—H1c16	0.96
C3—C9	1.530 (2)	C17—C21	1.526 (2)
C3—H1c3	0.96	C17—H1c17	0.96
C4—H1c4	0.96	C17—H2c17	0.96
C4—H2c4	0.96	C18—H1c18	0.96
C5—C7	1.400 (2)	C19—H1c19	0.96
C5—C19	1.398 (2)	C20—H1c20	0.96
C6—C16	1.373 (3)	C20—H2c20	0.96
C7—C15	1.514 (2)	C21—H1c21	0.96
C7—C18	1.392 (2)	C21—H2c21	0.96
			
C5—O1—H1	107.6 (15)	C13—C10—C18	122.66 (16)
C12—O2—H2	104.7 (16)	C9—C11—C21	110.60 (13)
C3—N1—C15	114.79 (12)	C9—C11—H1c11	109.47
C3—N1—C20	103.30 (11)	C9—C11—H2c11	109.47
C15—N1—C20	112.07 (11)	C21—C11—H1c11	109.47
C4—N2—C8	113.02 (11)	C21—C11—H2c11	109.47
C4—N2—C20	111.82 (11)	H1c11—C11—H2c11	108.32
C8—N2—C20	106.20 (11)	O2—C12—C2	121.15 (14)
C2—C1—C6	118.79 (16)	O2—C12—C14	118.45 (15)
C2—C1—H1c1	120.6	C2—C12—C14	120.40 (15)
C6—C1—H1c1	120.6	C10—C13—C19	118.45 (15)
C1—C2—C4	120.66 (14)	C10—C13—H1c13	120.78
C1—C2—C12	119.20 (14)	C19—C13—H1c13	120.78
C4—C2—C12	120.06 (14)	C12—C14—C16	120.23 (16)
N1—C3—C8	100.13 (11)	C12—C14—H1c14	119.89
N1—C3—C9	115.24 (12)	C16—C14—H1c14	119.89
N1—C3—H1c3	113.88	N1—C15—C7	112.94 (13)
C8—C3—C9	114.52 (12)	N1—C15—H1c15	109.47
C8—C3—H1c3	114.61	N1—C15—H2c15	109.47
C9—C3—H1c3	99.29	C7—C15—H1c15	109.47
N2—C4—C2	111.48 (12)	C7—C15—H2c15	109.47
N2—C4—H1c4	109.47	H1c15—C15—H2c15	105.76
N2—C4—H2c4	109.47	C6—C16—C14	118.49 (15)
C2—C4—H1c4	109.47	C6—C16—H1c16	120.75
C2—C4—H2c4	109.47	C14—C16—H1c16	120.75
H1c4—C4—H2c4	107.38	C8—C17—C21	111.23 (13)
O1—C5—C7	122.28 (13)	C8—C17—H1c17	109.47
O1—C5—C19	117.78 (15)	C8—C17—H2c17	109.47
C7—C5—C19	119.93 (15)	C21—C17—H1c17	109.47
F1—C6—C1	118.40 (16)	C21—C17—H2c17	109.47
F1—C6—C16	118.73 (14)	H1c17—C17—H2c17	107.66
C1—C6—C16	122.88 (16)	C7—C18—C10	119.35 (16)
C5—C7—C15	122.18 (14)	C7—C18—H1c18	120.33
C5—C7—C18	118.99 (14)	C10—C18—H1c18	120.32
C15—C7—C18	118.65 (14)	C5—C19—C13	120.61 (16)
N2—C8—C3	103.77 (11)	C5—C19—H1c19	119.7
N2—C8—C17	110.70 (12)	C13—C19—H1c19	119.7
N2—C8—H1c8	113.54	N1—C20—N2	105.82 (11)
C3—C8—C17	112.67 (12)	N1—C20—H1c20	109.47
C3—C8—H1c8	111.6	N1—C20—H2c20	109.47
C17—C8—H1c8	104.79	N2—C20—H1c20	109.47
C3—C9—C11	113.83 (13)	N2—C20—H2c20	109.47
C3—C9—H1c9	109.47	H1c20—C20—H2c20	112.89
C3—C9—H2c9	109.47	C11—C21—C17	109.50 (13)
C11—C9—H1c9	109.47	C11—C21—H1c21	109.47
C11—C9—H2c9	109.47	C11—C21—H2c21	109.47
H1c9—C9—H2c9	104.73	C17—C21—H1c21	109.47
F2—C10—C13	118.53 (14)	C17—C21—H2c21	109.47
F2—C10—C18	118.81 (16)	H1c21—C21—H2c21	109.45

### Hydrogen-bond geometry (Å, º)

**Table:**

*D*—H···*A*	*D*—H	H···*A*	*D*···*A*	*D*—H···*A*
O1—H1···N1	0.85 (2)	1.89 (2)	2.6540 (17)	148 (2)
O2—H2···N2	0.85 (2)	1.89 (2)	2.6741 (18)	152 (2)
O1—H1···C15	0.85 (2)	2.45 (2)	2.937 (2)	117.4 (18)
O2—H2···C4	0.85 (2)	2.35 (2)	2.867 (2)	119.3 (19)
C13—H1C13···F2^i^	0.96	2.43	3.2645 (19)	145
C17—H2C17···F2^ii^	0.96	2.54	3.356 (2)	142

Symmetry codes: (i) −*x*, −*y*, *z*+1/2; (ii) *x*+1/2, −*y*+1/2, *z*+1.
